# Pharmacological manipulation of DNA methylation normalizes maternal behavior, DNA methylation, and gene expression in dams with a history of maltreatment

**DOI:** 10.1038/s41598-019-46539-4

**Published:** 2019-07-16

**Authors:** Samantha M. Keller, Tiffany S. Doherty, Tania L. Roth

**Affiliations:** Department of Psychological and Brain Sciences, University of Delaware, Newark, DE 19716 USA

**Keywords:** Neuroscience, Epigenetics and behaviour

## Abstract

The quality of parental care received during development profoundly influences an individual’s phenotype, including that of maternal behavior. We previously found that female rats with a history of maltreatment during infancy mistreat their own offspring. One proposed mechanism through which early-life experiences influence behavior is via epigenetic modifications. Indeed, our lab has identified a number of brain epigenetic alterations in female rats with a history of maltreatment. Here we sought to investigate the role of DNA methylation in aberrant maternal behavior. We administered zebularine, a drug known to alter DNA methylation, to dams exposed during infancy to the scarcity-adversity model of low nesting resources, and then characterized the quality of their care towards their offspring. First, we replicate that dams with a history of maltreatment mistreat their own offspring. Second, we show that maltreated-dams treated with zebularine exhibit lower levels of adverse care toward their offspring. Third, we show that administration of zebularine in control dams (history of nurturing care) enhances levels of adverse care. Lastly, we show altered methylation and gene expression in maltreated dams normalized by zebularine. These findings lend support to the hypothesis that epigenetic alterations resulting from maltreatment causally relate to behavioral outcomes.

## Introduction

Infant experiences with a caregiver have lifelong behavioral consequences and the mechanisms through which these early-life experiences are capable of inducing long-term effects on phenotype continue to be elucidated^1–5^. Epigenetic alterations offer one potential mechanism through which experiences in infancy can perpetuate their consequences throughout the lifespan^2,6–16^. For example, experiencing adverse maternal care induces both transient and long-term modifications to the epigenome^2,9,17–23^. Epigenetic mechanisms, such as DNA methylation and posttranslational histone modifications, are capable of influencing gene expression without altering the underlying genomic sequence. DNA methylation, or the addition of methyl groups to cytosine residues on DNA, typically represses the expression of genes^24–26^. These epigenetic modifications can have functional implications by altering levels of gene expression and in turn protein products, in specific brain regions that control behavior.

Maternal behavior is a complex behavior requiring the recruitment of multiple brain regions including the nucleus accumbens (NAC)^27–29^, bed nucleus of the stria terminalis (BNST)^30–35^, ventral tegmental area (VTA)^34,36–38^, prefrontal cortex (PFC)^38–40^, amygdala^41–45^, and medial preoptic area (MPOA)^30,35,39,46,47^. In this circuit, hormones including estrogen act on the MPOA to stimulate maternal behavior^47,48^. The MPOA is then primed to become active in response to pup stimuli. The PFC and amygdala are involved in processing sensory information, such as pup scent, which elicit maternal responsiveness^49^. The MPOA in turn projects to the VTA, which provides dopaminergic input to the NAC. This projection is important for the rewarding component of pup interactions^50^. Dysregulation within this circuitry can lead to altered or impaired maternal responsiveness^49^, and epigenetic modifications within this circuit are one potential mechanism through which dysregulation could occur. Indeed, experience-driven alterations in DNA methylation in maternal circuitry can influence maternal behavior via altered functioning in these brain regions^7,51^.

Our lab employs the scarcity-adversity model of low nesting resources, a validated rodent model of caregiver maltreatment^52^. The scarcity-adversity model of low nesting resources is a within litter paradigm whereby 1/3 of the litter is left in the home cage with the biological dam, 1/3 of the litter is removed from the home cage and placed in another environment for 30 minutes with a dam that provides nurturing care, and the remaining 1/3 of the litter is removed from the home cage and placed in another environment for 30 minutes with a dam that provides adverse care. Infant rats are exposed to these caregiving conditions daily during the first seven days of life. Utilizing this model, our lab has previously identified altered gene expression and DNA methylation in some of the brain regions controlling maternal behavior^2,18,53^. Coinciding with altered patterns of DNA methylation, our lab has likewise found aberrant maternal behavior (more adverse and less nurturing care) in females subjected to maltreatment^2^, consistent with studies in humans finding disrupted maternal behavior (e.g. increased hostility and reduced warmth toward children, impaired mother-child bonding, and increased use of physical punishment) in women that experienced childhood abuse^54–59^. Maternal behavior is an intergenerational behavior, as the quality of maternal care a female experiences influences the quality of care she will give her own offspring^2,58,60–62^. Therefore, it is important to establish the neurobiological underpinnings of aberrant maternal behavior and explore treatments that can improve maternal behavior to prevent the perpetuation of poor maternal care across generations.

In previous work, we demonstrated the ability of the epigenome-altering drug, zebularine, to reverse maltreatment-induced DNA methylation and expression of the brain-derived neurotrophic factor (*Bdnf*) gene in the adult PFC^2^ and alter some adult behavioral outcomes^63^. Based upon this, in the current study, we sought to assess the ability of zebularine to rectify consequences of maltreatment on maternal behavior when administered to adult dams. To examine the neurobiological underpinnings of maternal behavior deficits in maltreated animals, we assayed DNA methylation and gene expression within the MPOA due to this brain region’s critical role in maternal behavior^30,34,35,44,49^.

## Results

### Infant manipulations

**Figure 1:**
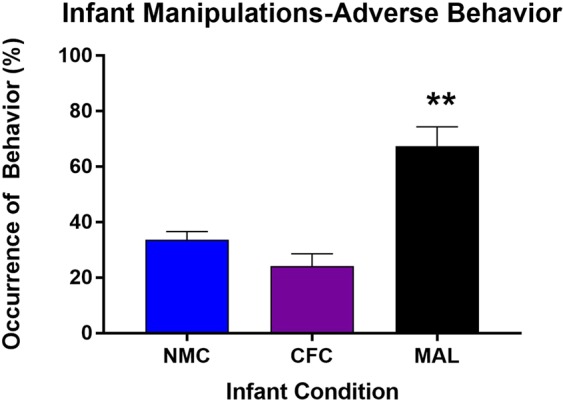
Pups in the maltreatment condition incurred a higher prevalence of adverse behaviors from the caregiver relative to pups placed in the cross-foster and normal maternal care conditions. These data are presented as percentage of occurrence of behavior in 5-minute time bins across the 30 minute behavioral recordings (averaged across the 7 days). n = 5 litters; **Denotes *p* < 0.01, comparison is the maltreatment group versus both the normal care and cross-foster care groups. NMC = normal maternal care; CFC = cross-foster care; MAL = maltreatment.

A one-way ANOVA performed on adverse behaviors observed across our infant manipulations revealed a main effect of infant condition (*F*_(2,12)_ = 20.17, *p* = 0.0001, Cohen’s *d* = 3.176; Fig. 1). Post-hoc comparisons showed that animals in the maltreatment condition experienced significantly more adverse behaviors relative to those in the normal (*p* = 0.0013) and cross-foster (*p* = 0.0002) care conditions. We did not find any differences in the levels of adverse care between the cross-foster and normal care conditions (*p* = 0.4145). These results are consistent with previous reports employing the scarcity-adversity model of low nesting resources^1,2,18^. These data validate the efficacy of our model to experimentally induce an adverse caregiving environment.

### Trivers–willard effect

**Figure 2:**
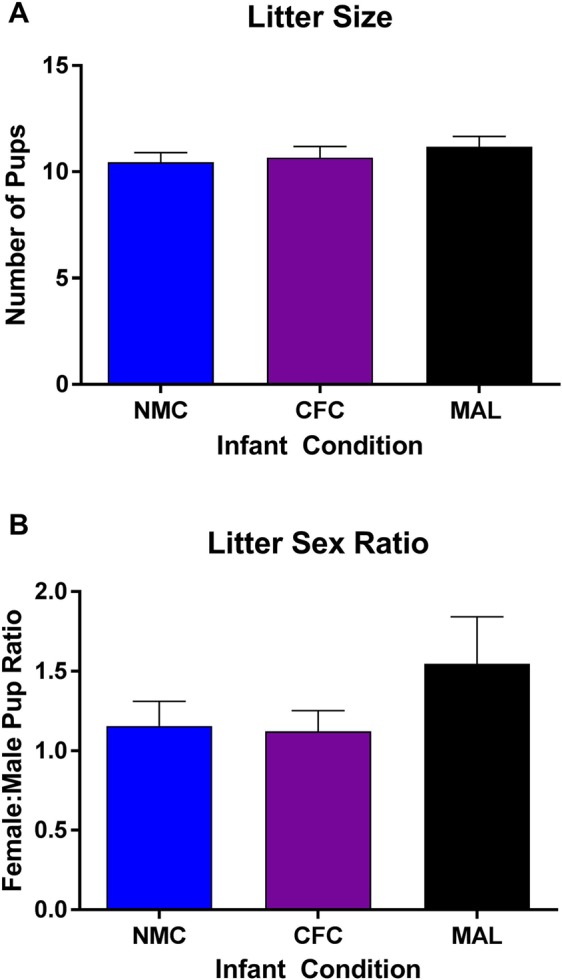
No differences were found in litter size (**A**) or the female:male pup ratio (**B**) as a result of infant caregiver condition. n = 22–24/group. NMC = normal maternal care; CFC = cross-foster care; MAL = maltreatment.

To examine the presence of the Trivers–Willard effect, which predicts that females with a history of stress will have a higher female:male pup ratio^64^, pups were sexed and counted. We did not find a significant difference in the litter size (*F*_(2,65)_ = 0.5636, *p* = 0.5719, Fig. 2A) or female:male pup ratio (*F*_(2,65)_ = 1.34, *p* = 0.2691, Fig. 2B) as a result of infant condition. These data indicate that within the scarcity-adversity model of low nesting resources as employed here, dams with a history of maltreatment do not show altered litter compositions compared to control dams.

### Adult maternal behavior

**Figure 3:**
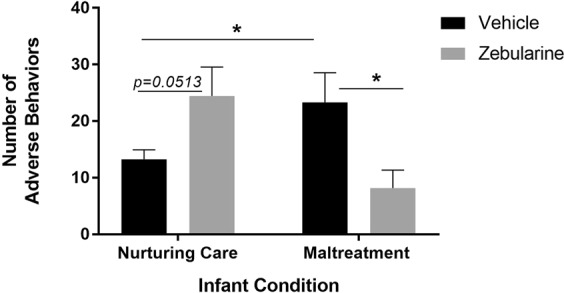
Animals with a history of maltreatment exhibited more adverse caregiving behaviors toward their pups as compared to dams without a history of maltreatment (i.e. dams with a history of nurturing infant care). Treatment with zebularine significantly reduced levels of adverse behavior exhibited toward offspring in previously maltreated dams. Zebularine treatment disturbed behavior in dams without a history of maltreatment such that drug-treated dams exhibited higher levels of adverse behavior toward offspring relative to vehicle-treated controls. n = 10–23/group; *denotes *p* < 0.05, comparisons indicated by black lines.

No significant differences in maternal behavior were found between subjects in the cross-foster and normal maternal care vehicle groups (*t*_(19)_ = 0.5541, *p* = 0.5927) or the cross-foster and normal maternal care zebularine groups (*t*_(21)_ = 1.071, *p* = 0.2962), therefore the nurturing care vehicle groups were collapsed and the nurturing care zebularine groups were collapsed to increase statistical power. A two-way ANOVA performed on the number of adverse behaviors performed by dams demonstrated an infant condition X drug treatment interaction (*F*_(1,60)_ = 8.036, *p* = 0.0062, Cohen’s *d* = 1.126, Fig. 3). Consistent with our previous finding^2^, post-hoc analyses revealed that females with a history of maltreatment (i.e. maltreatment-vehicle group) performed more adverse behaviors toward their offspring relative to animals with a history of nurturing care (i.e. nurturing-vehicle group) (*t*_(29)_ = 2.315, *p* = 0.0279). There was a significant difference between the maltreatment group administered zebularine versus the vehicle-treated maltreatment group, suggesting that zebularine rescued maltreatment-induced aberrations in maternal behavior (*t*_(18)_ = 2.466, *p* = 0.0239).

Treatment with zebularine disrupted maternal care in females without a history of maltreatment (i.e. controls), such that drug-treated controls showed numerically more adverse behaviors than their vehicle-treated counterparts (*t*_(42)_ = 2.006, *p* = 0.0513). No significant differences were found between the zebularine-treated dams with a history of nurturing care versus the maltreated dams given vehicle (*p* = 0.8927), or the vehicle-treated nurturing care group versus the zebularine-treated maltreatment group (*p* = 0.1332). Taken together, these data indicate that zebularine normalizes maternal behavior in dams with a history of maltreatment while disturbing maternal behavior in dams with a history of nurturing care in infancy.

### DNA methylation

**Figure 4:**
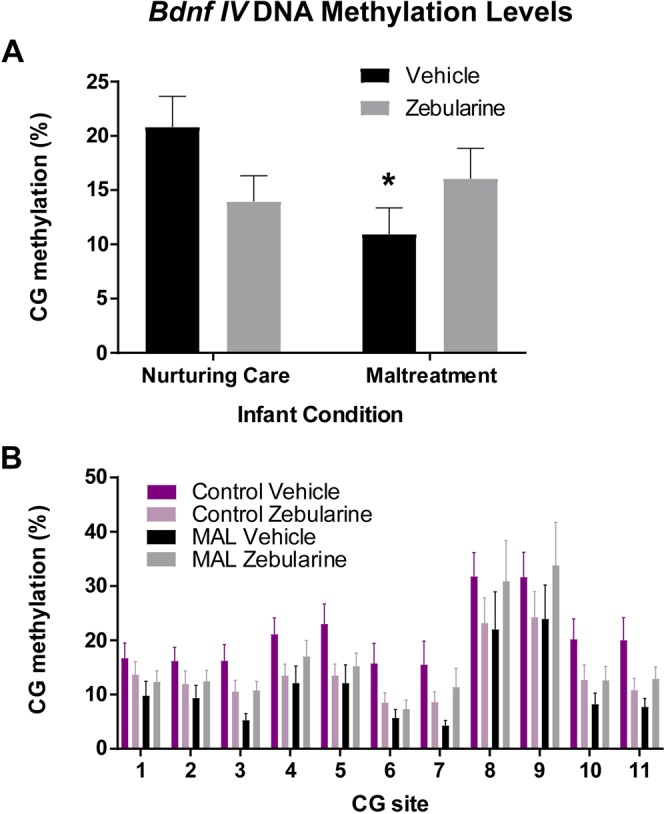
DNA methylation of *Bdnf* exon *IV* (average levels across the 11 CG sites represented in panel A, site-specific levels represented in panel B) was reduced in vehicle-treated animals with a history of maltreatment. Administration of zebularine in adulthood normalized DNA methylation in these dams. n = 10–23/group; *Denotes *p* < 0.05, comparison is the maltreatment vehicle group versus the control vehicle group.

No significant differences in average levels of methylation were detected between control vehicle groups (cross-foster vs. normal care, *p* = 0.9407) or control zebularine groups (cross-foster vs. normal care, *p* = 0.4081), thus control groups administered the same treatment were collapsed. Analysis of *Bdnf* methylation of exon *IV* DNA revealed a significant infant condition X drug interaction (*F*_(1,63)_ = 4.088, *p* = 0.0474, Cohen’s *d* = 0.77, Fig. 4A). Post-hoc analyses revealed that animals with a history of maltreatment displayed significantly reduced levels of methylation relative to control animals delivered vehicle (*p* = 0.034). However, maltreated animals administered zebularine did not show different levels of methylation relative to controls (*p* = 0.3053), suggesting that zebularine was able to normalize methylation levels in animals with a history of maltreatment. While numerically different levels of methylation were observed between control animals administered zebularine as compared to vehicle, this relationship did not reach statistical significance (*p* = 0.0715).

Two-way ANOVAs were performed on each of the 11 CG sites sequenced for *Bdnf IV* (Fig. 4B). There was an infant condition X drug interaction at CG site 3 (*F*_(1,63)_ = 4.13, *p* = 0.0464, Cohen’s *d* = 0.774), with post-hoc analyses revealing that animals with a history of maltreatment displayed significantly reduced levels of methylation relative to control animals delivered vehicle (*p* = 0.0312). There was also an infant condition X drug interaction at CG sites 4 (*F*_(1,63)_ = 4.153, *p* = 0.0458, Cohen’s *d* = 0.776) and 11 (*F*_(1,62)_ = 4.043, *p* = 0.0487, Cohen’s *d* = 0.773), however post hoc analyses failed to meet significance for either site.

**Figure 5:**
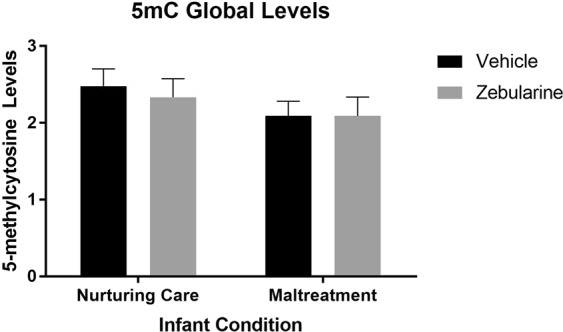
Percent global DNA methylation levels in the MPOA. n = 6–14/group.

We also conducted an assay using a partial set of the same DNA samples (n = 6–14, randomly chosen) to assess global 5mc levels in the MPOA (Fig. 5). There were no effects of drug treatment (*F*_(1,36)_ = 0.06781, *p* = 0.796), infant condition (*F*_(1,36)_ = 1.1323, *p* = 0.2577), nor an interaction effect (*F*_(1,36)_ = 0.07113, *p* = 0.7912).

### Gene expression

**Figure 6:**
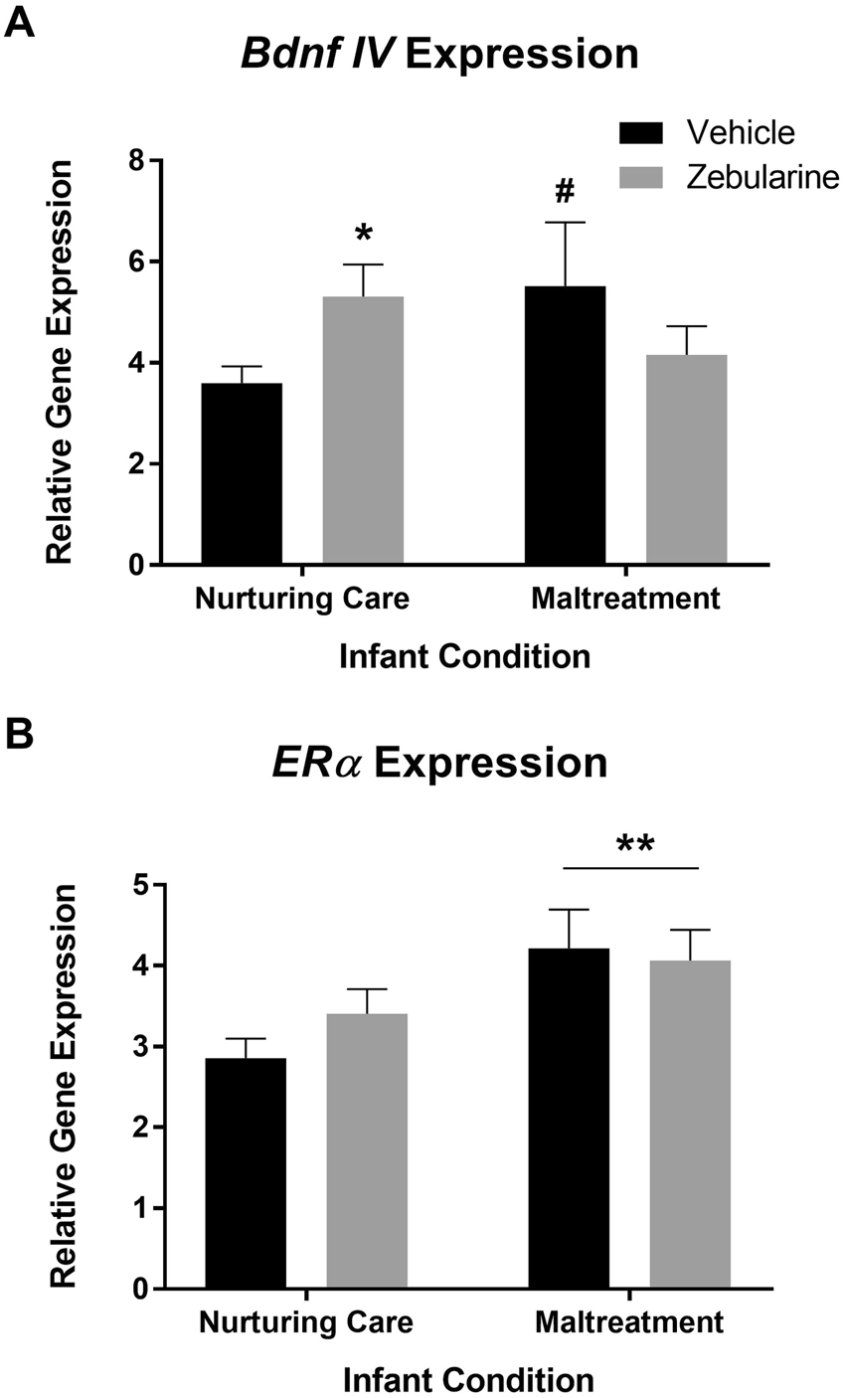
Animals with a history of maltreatment exhibited marginally elevated levels of *Bdnf* exon *IV* expression relative to animals with a history of nurturing care in infancy. Treatment with zebularine normalized levels of *Bdnf IV* expression in maltreated dams, however, this same treatment increased levels of *Bdnf IV* expression in animals with a history of nurturing maternal care (**A**). Maltreated dams exhibited increased *ERα* expression regardless of drug treatment (**B**). n = 10–23/group; *Denotes *p* < 0.05, comparison is the vehicle and zebularine nurturing care groups; **Denotes p < 0.01, comparison is maltreatment versus controls; ^#^Denotes *p* < 0.06, comparison is the maltreatment vehicle group versus the control vehicle group.

Control groups administered vehicle were collapsed and control groups administered zebularine were collapsed, as no significant group differences in gene expression were found (*p*’s > 0.05). No significant effects of drug treatment, infant condition, nor interactions were found for expression of *fos*, *DNMT1*, *DNMT3a*, *Bdnf* exons *I* and *IX*, *Drd1*, *Oprm*, *Oxtr*, *Crfr1*, or *GR* (*p*’s > 0.05, Table 1). For *DNMT1*, an ANOVA revealed a marginally significant main effect of infant condition (*F*_(1,61)_ = 3.903, *p* = 0.0527, Cohen’s *d* = 0.753), however, post-hoc analyses failed to meet significance (nurturing care versus maltreatment vehicle groups, *p* = 0.0852). With regard to *Bdnf IV* expression, a two-way ANOVA revealed a significant infant condition X drug interaction (*F*_(1,62)_ = 4.848, *p* = 0.0314, Cohen’s *d* = 0.839, Fig. 6A). Maltreated dams exhibited higher levels of *Bdnf* expression relative to control vehicle-treated dams (*p* = 0.0579), but no difference was found between maltreated dams given zebularine relative to vehicle-treated control animals (*p* = 0.372). A significant difference was found between control vehicle and zebularine groups, with zebularine increasing expression levels in control animals (*p* = 0.0363).

**Table 1:** This table contains the group means with standard errors in parentheses for relative gene expression (A) and statistical analyses (B) for all genes examined via real-time PCR that did not exhibit any statistically significant effects. n = 6–23/group.

Gene	Nurturing Care Vehicle	Nurturing Care Zebularine	Maltreatment Vehicle	Maltreatment Zebularine
*fos*	2.19 (0.22)	2.24 (0.20)	2.07 (0.20)	2.25 (0.25)
*DNMT1*	2.07 (0.27)	1.89 (0.12)	2.95 (0.66)	2.13 (0.27)
*DNMT3a*	2.15 (0.18)	1.91 (0.09)	2.37 (0.25)	2.20 (0.23)
*Bdnf I*	2.72 (0.25)	2.95 (0.15)	3.21 (0.35)	2.93 (0.32)
*Bdnf IX*	25.52 (4.33)	32.03 (5.60)	26.68 (6.29)	17.51 (3.18)
*Drd*	3.55 (0.48)	3.94 (0.54)	4.49 (0.90)	3.83 (0.65)
*Oprm*	1.73 (0.09)	1.82 (0.09)	2.10 (0.22)	1.87 (0.19)
*Oxtr*	1.79 (0.085)	1.76 (0.08)	1.89 (0.18)	1.75 (0.10)
*Crfr1*	2.81 (0.22)	3.01 (0.15)	3.06 (0.24)	2.76 (0.37)
*GR*	2.09 (0.15)	2.09 (0.11)	2.21 (0.21)	2.47 (0.27)
**Gene**	**Infant Condition**	**Drug Treatment**	**Interaction**	
*fos*	*F*_(1,62)_ = 0.06542, *p* = 0.7990	*F*_(1,62)_ = 0.2271, *p* = 0.6354	*F*_(1,62)_ = 0.07647, *p* = 0.7831	
*DNMT1*	*F*_(1,61)_ = 3.903, *p* = 0.0527	*F*_(1,61)_ = 0.5126, *p* = 0.4768	*F*_(1,61)_ = 0.187, *p* = 0.6670	
*DNMT3a*	*F*_(1,63)_ = 1.829, *p* = 0.1811	*F*_(1,63)_ = 1.236, *p* = 0.2705	*F*_(1,62)_ = 0.5444, *p* = 0.4634	
*Bdnf I*	*F*_(1,64)_ = 0.7771, *p* = 0.3813	*F*_(1,64)_ = 0.01114, *p* = 0.9163	*F*_(1,64)_ = 0.9374, *p* = 0.3366	
*Bdnf IX*	*F*_(1,62)_ = 1.294, *p* = 0.2597	*F*_(1,62)_ = 0.05109, *p* = 0.8219	*F*_(1,62)_ = 1.78, *p* = 0.1871	
*Drd1*	*F*_(1,60)_ = 0.4011, *p* = 0.5289	*F*_(1,60)_ = 0.04356, *p* = 0.8354	*F*_(1,60)_ = 0.6327, *p* = 0.4295	
*Oprm*	*F*_(1,62)_ = 2.31, *p* = 0.1336	*F*_(1,62)_ = 0.2276, *p* = 0.6350	*F*_(1,62)_ = 1.386, *p* = 0.2436	
*Oxtr*	*F*_(1,64)_ = 0.1464, *p* = 0.7033	*F*_(1,64)_ = 0.6406, *p* = 0.4264	*F*_(1,64)_ = 0.2471, *p* = 0.6208	
*Crfr1*	*F*_(1,41)_ = 0.0001146, *p* = 0.9915	*F*_(1,41)_ = 0.03667, *p* = 0.8491	*F*_(1,41)_ = 0.9792, *p* = 0.3282	
*GR*	*F*_(1,62)_ = 1.997, *p* = 0.1626	*F*_(1,62)_ = 0.5444, *p* = 0.4634	*F*_(1,62)_ = 0.5065, *p* = 0.4793

A significant effect of infant condition was found for *ERα* expression (*F*_(1,62)_ = 8.2, *p* = 0.0057, Cohen’s *d* = 1.091, Fig. 6B), such that maltreated animals administered zebularine or vehicle showed elevated levels of *ERα* expression relative to control animals. No significant effects of drug treatment or a drug X infant condition interaction were detected (*p*’s > 0.05). Taken together with the methylation data, results are consistent with the notion that maltreatment and zebularine have gene-specific effects.

## Discussion

We replicated our previous finding that dams with a history of maltreatment mistreat their own offspring^2^. Further, we found that daily administration of zebularine at a dose previously shown to rescue aberrant DNA methylation and gene expression^2^ and forced swim behavior^63^ normalized maternal behavior in maltreated dams. Interestingly, this drug disturbed maternal behavior in animals without of a history of maltreatment such that these animals displayed enhanced levels of adverse behaviors toward offspring. We likewise found that zebularine was able to normalize *Bdnf* exon *IV* levels of methylation and gene expression. Consistent with the behavioral data, zebularine administration disturbed levels of *Bdnf* gene expression in animals with a history of nurturing maternal care in infancy. These data suggest that the effects of zebularine are specific to caregiving history, as the drug elicited opposite effects in animals with a history of maltreatment relative to animals with no history of maltreatment.

While it may seem perplexing that zebularine had contrasting effects on dams dependent on their early-life history, our lab has previously identified a number of differences within the epigenome resulting from exposure to our maltreatment paradigm^2,9,17–20,53^. These differences are widespread; we have discovered maltreatment-induced changes in methylation in the PFC^2,17,18^, amygdala^20^, and hippocampus^20^. Thus, it seems plausible that the divergent effects of the drug could be a result of the existing epigenetic differences in animals with different caregiving histories. Presumably, zebularine normalized aberrant methylation in dams with a history of maltreatment, and this in turn normalized their maternal behavior. Indeed, we found methylation of the *Bdnf* gene to be disrupted in maltreated animals. On the other hand, zebularine presumably disrupted normative methylation patterns in dams without a history of maltreatment which, in turn, produced disruptions in their maternal behavior. Consistent with our results, several studies have previously found behavioral disruptions in non-stressed animals administered zebularine^65–68^. These results hint heavily at the causal nature of the relationship between the epigenome and behavioral phenotypes. Our data also argue that future work is warranted to discern the conditions under which zebularine has beneficial as opposed to harmful impacts on behavior.

There is a precedent in the literature for other epigenome-modifying drugs to alter maternal responsiveness in female rodents. In a maternal sensitization paradigm, female mice treated with a histone deacetylase inhibitor showed increased maternal responsiveness toward pups^69,70^. In this study, the expression of genes known to be involved in maternal behavior, including estrogen receptor beta (*ERβ*) was altered for 30 days following the sensitization paradigm^70^, suggesting that the facilitatory effects of this drug on maternal behavior can be long-lasting. Additionally, maternal behavior has been improved by administration of drugs whose main target is not the epigenome, such as those that alter reward circuitry. For example, increasing levels of dopamine pharmacologically was found to increase levels of licking and grooming toward offspring in dams that received low levels of licking and grooming as infants^71^. However, to the knowledge of the authors this is the first time that an epigenome-modifying drug has been utilized to rectify maltreatment-induced aberrations in maternal behavior in adult animals.

Experiencing different types of early-life stress elicits diverse biological and behavioral outcomes^13,72–74^. It is unknown if behavioral consequences resulting from other types of early-life stress, such as maternal separation or prenatal stress, could be rectified by altering DNA methylation by the same strategy employed here. Additionally, our study focused on a female-specific behavior, and as such it is unclear if this drug would likewise ameliorate behavioral consequences of maltreatment in male subjects. For example, male rats subjected to the scarcity-adversity model of low nesting resources demonstrate deficits in fear extinction that are not observed in females exposed to the model^1^. Sex differences exist throughout the epigenome^75–77^ and in levels of epigenetic regulators^78,79^, so it is possible that epigenome-modifying treatments would not be equally effective in male and female subjects. It should be noted however that previous work from our lab established an ability for zebularine administered at the same dose as this study to rescue maltreatment-induced DNA methylation and gene expression in the PFC of both female and male subjects, which would suggest that zebularine would likewise be efficacious for behavioral deficits elicited by maltreatment in males^2^. While we examined behavior at a time point when maltreatment-induced DNA methylation is known to be rescued by zebularine treatment (i.e. 24 hours after a week of daily infusions), looking at the ability for zebularine to change behavior over the course of the seven day infusion regimen would also be an interesting future direction.

Our data lend support to the hypothesis that the epigenetic consequences of stress are causally linked to the behavioral outcomes. Further, these data suggest a role of *Bdnf* in maternal behavior, as we found *Bdnf* methylation and gene expression data to match the behavioral data (i.e. zebularine disturbed *Bdnf* levels and behavior in normal animals while rescuing behavior and *Bdnf* levels in maltreated animals). Numerous studies have found links between early life stress and *Bdnf* methylation and expression^2,9,20,80–82^ and early life stress and altered maternal behavior^2,56–58^, but the direct relationship between maternal behavior and *Bdnf* has been largely unexplored. One study has provided evidence of correlations between *Bdnf* DNA methylation and neural activity associated with maternal response to child stimuli^83^. While psychiatric disorders such as depression and posttraumatic stress disorder have been linked with low levels of *Bdnf* expression in certain brain regions such as the hippocampus^84–86^, elevated levels of *Bdnf* have likewise been linked with deleterious outcomes^87–89^. It is possible that aberrant levels of *Bdnf* expression in either direction (i.e. elevated or reduced as compared to normative levels) could induce adverse outcomes. We speculate that, because *Bdnf* is associated with plasticity, aberrant *Bdnf* levels could interfere with the plasticity that typically occurs in dams to drive maternal behavior. Consistent with this notion, our study found levels of *ERα* to be altered in maltreated dams, and *ERα* is one target of plasticity in the maternal brain^32,90^. Further study is necessary to elucidate the contribution of *Bdnf* to maternal behavior.

Because the MPOA is a heterogeneous nucleus with varied neuronal projections^91,92^, future work is needed to discern the precise neurobiological pattern of gene expression altered by maltreatment and zebularine treatment and the contribution of these sub-regions to maternal behavior in the scarcity-adversity model of low nesting resources. Though certain genes, including *ERα*, have been found to be altered as a result of early life experience in only select sub-regions of the MPOA^93^, our methods do not provide this level of detail. One seemingly counterintuitive finding from our study was the increased expression of *ERα* in MPOA of the maltreatment groups. This is in contrast to other reports that found increased *ERα* expression in the MPOA of animals that received nurturing care during early postnatal life^7,90,93^. Direct comparison between our study and others is difficult because of the different early-life paradigms and measures of maternal behavior employed, which could be responsible for the different outcomes^10,52^. Perhaps, elevated levels of *ERα* expression in the MPOA of maltreated dams serve a compensatory function, as *ERα* is a transcription factor that drives the expression of other genes^94–97^.

While we found *Bdnf* methylation to be normalized in the brain of maltreated dams administered zebularine, future work is needed to establish which other genes could be underlying the observed behavioral effects of zebularine. It is important to note that, similar to our data here, other reports show selective drug-induced changes in expression of genes involved in maternal behavior associated with enhanced maternal responsiveness^70^. For example, in the previously mentioned study that observed changes in *ERβ* expression after exposure to a histone deacetylase inhibitor, no changes in *Oxtr* expression were detected^70^. Such data support the idea that altering methylation and expression of select genes is sufficient to alter maternal responsiveness. However, the impact of epigenome-modifying drugs are time specific^98–100^. Since we have data from only one time point (i.e. 24 hours after the final of 7 daily infusions), it is unclear whether we would have seen a different pattern of gene expression and DNA methylation at other time points. Additional time points as well as addressing the circuit-level alterations responsible for the deficits seen in maternal behavior that zebularine is capable of modulating are important avenues for future research.

Zebularine cannot cross the blood-brain barrier and therefore needs to be administered centrally^101^. Further work is certainly warranted to determine whether less invasive treatments such as exercise, diet, and social enrichment, all measures that can easily be employed in humans, could have facilitatory effects on maternal behavior through altering the epigenome. The data reported here help to construct the necessary foundation for such efforts, as well as those showing that exercise^102–104^, diet^105–107^, and social interaction^108–110^ have lasting influences on the epigenome. Continued exploration of factors that can affect behavioral outcomes associated with early adversity holds promise of knowledge that can then be leveraged in the development of treatments and/or interventions for humans affected by early adversity. This is especially critical given that the impact of maltreatment on brain and behavior is multigenerational. One example of this comes from a recent study that found reduced cortical gray matter volume in the brains of offspring of women that experienced maltreatment in childhood^111^. It is thus important to establish treatments aimed at maternal behavior and the associated neurobiological deficits to not just improve outcomes for those directly exposed to adversity but the outcomes for following generations as well.

Overall, the finding that targeting the epigenome was successful in attenuating poor maternal behavior and aberrant DNA methylation and gene expression is an exciting step forward in the literature. These data confirm the utility of a rodent model to study the behavioral phenomenon of multigenerational patterns of parenting. They then further provide support for studying the relationship between maltreatment-induced epigenetic modifications and perpetuated patterns of maternal maltreatment of offspring. Finally, they offer insight into the potential of exploiting that relationship to subvert the often tragic outcomes of adversity.

## Methods

### Subjects

**Figure 7:**
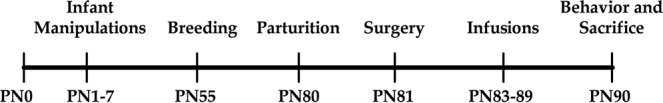
This figure depicts an approximate timeline of experimental procedures that occurred to our experimental subjects. First, animals were exposed to the infant caregiver manipulations from PN1-7. Next, they were bred with naïve breeder males and allowed to give birth to their own litter. One day following parturition, stereotaxic surgery to implant a chronic cannula into the left lateral ventricle was conducted and subjects were allowed one day of recovery. After recovery, infusions of either zebularine or vehicle were administered daily for seven days. One day after the final infusion, a behavioral video was collected and brains were harvested from the subjects.

All animal procedures were conducted following approval by the University of Delaware Institutional Animal Care and Use committee using NIH established guidelines. All experiments were performed in accordance with relevant guidelines and regulations. This study utilized Long-Evans rats that were bred in house. Dams were maintained on a 12 hour light/dark cycle and were given *ad libitum* access to food and water. Postnatal day (PN) 0 was classified as the day of parturition. Figure 7 provides an approximate timeline of experimental procedures performed in this study.

### Caregiving manipulations

Rodent pups were exposed to the scarcity adversity model of low nesting resources for 30 minutes per day from PN 1–7^1,2,18–20,52,82^. This model employs a within litter design whereby 1/3 of the litter is dedicated to the maltreatment condition, 1/3 of the litter is dedicated to the cross-foster care condition, and 1/3 of the litter receives normal maternal care. For the maltreatment condition, pups were exposed to another dam with limited nesting resources (~100 ml) in a novel environment. Dams were matched for postpartum age and diet to the biological dam of the experimental litter, as pups are unable to distinguish between their biological dam and a diet-matched dam^112^. In the cross-foster care condition, pups were also exposed to another dam in a novel environment. However, this dam had been given ample nesting resources (2–3 cm layer) and is familiar with the environment (i.e. had habituated for one hour). In the normal maternal care condition, each day the pups were marked with a nontoxic Sharpie® marker, weighed, and subsequently returned to the home cage with their biological mother. When possible, equal numbers of male and female pups were placed into each caregiver condition. However, at least one male and one female pup were placed into each of the 3 experimental groups. Caregiving behavior in each of these conditions was recorded. A subset of 5 of the 13 litters from which experimental subjects were taken was scored to confirm the replicability of this model (i.e. increased levels of adverse behavior by the caregiver during the maltreatment condition relative to the two control conditions). Videos were coded for adverse (roughly handling, dropping, dragging, stepping on, or actively avoiding pups) behaviors in five-minute time bins. The resulting scores for each caregiving behavior were then averaged across the seven sessions for statistical analysis (thus data shown in Fig. 1 are comprised of caregiving behavior from PN 1–7).

At the time of weaning, male and female offspring were separated and only female offspring were utilized for the duration of the study. Males were used for other experiments in our laboratory. Female subjects were placed into cages of two or three animals from the same infant condition. When female rodents exposed to these infant manipulations reached adulthood (around PN55), they were be bred with naïve breeder males and permitted to give birth. After the female had successfully bred with the male (i.e. a sperm plug was found), animals were single-housed and remained undisturbed until one day following parturition. To examine the presence of the Trivers–Willard effect pups were sexed and counted^64^.

### Stereotaxic surgery and drug infusions

One day after parturition, stereotaxic surgery was performed to implant a cannula into the left lateral ventricle following a protocol similar to one used previously by our lab^2^. To induce anesthesia, dams were placed in an induction chamber containing 5% isoflurane in oxygen. Once anesthesia was induced, animals were administered 2 mL of sterile saline and 0.03 mg/kg buprenorphine. The dam was then placed into a stereotaxic frame. Anesthesia was maintained using 2–3% isoflurane in oxygen and a stainless steel guide cannula (22 gauge, 8 mm length, Plastics One Inc., Roanoke, VA) was implanted into the left lateral ventricle (1.5 mm posterior, 2.0 mm lateral, and 3.0 mm ventral relative to bregma). At the time of surgery, cannula placement was verified using gravitational saline letdown as has been done in previous reports^113^. A dummy cannula extending 1 mm beyond the guide cannula was inserted into the guide cannula upon cessation of surgery to prevent cannula blockage. While the dam was undergoing surgery, her pups were left in the home cage on a heating pad and monitored. Dams were allowed one day of recovery after surgery during which they were left undisturbed and monitored to ensure appropriate recovery (e.g. maintaining weight and grooming properly).

Following recovery, daily infusions of zebularine or vehicle were performed. Zebularine is a cytidine analog known to incorporate into DNA and consequently alters DNA methylation^114^. This drug is known to alter levels of DNA methylation when administered to adult rats^2,65,66,68^. We selected a drug dose and treatment regimen shown to reverse aberrant DNA methylation and gene expression levels^2^ as well as reverse maltreatment-induced aberrations in forced swim behavior^63^. Specifically, zebularine (600 ng/μl in 10% DMSO, 2 μl volume, infusion rate of 1 μl/min) was administered once daily for seven days. Vehicle was comprised of 10% DMSO in sterile saline. Each experimental group contained between 10–12 subjects.

### Adult behavior

A 30 minute behavioral recording was collected 24 hours following the final infusion, as this is the same time point we have observed an effect of zebularine on methylation and gene expression^2^. Recordings were later coded offline for adverse (roughly handling, dropping, dragging, stepping on, and avoiding the pups) maternal behaviors by scorers blind to experimental conditions. Because a previous report from our lab found enhanced levels of adverse behaviors performed toward offspring in dams with a history of maltreatment^2^, the total number of adverse behaviors conducted throughout the 30 minute recording were tallied. This method of recording each bout of a behavior has been used by others to probe for differences in maternal care in dams with a history of early-life stress^115^, providing more resolution that can be lost when collapsing behaviors across time bins (as we have commonly done).

### DNA methylation

Immediately after the behavioral recording was collected 24 hours following the final infusion, dams were sacrificed and brains were harvested. Brains were flash-frozen in isopentane and stored at −80 °C until later processing. Brains were sliced at 250 μm in a cryostat at −12 °C. Cannula placement in the left lateral ventricle was visually confirmed during brain slicing. Tissue was dissected from the MPOA over dry ice using stereotaxic coordinates obtained from a brain atlas (AP + 0.12 mm through −0.75 mm relative to bregma)^116^. DNA and RNA were extracted from the MPOA using the Qiagen AllPrep DNA/RNA Mini Kit. To analyze nucleic acid quality and concentration, spectrophotometry was conducted (NanoDrop 2000). DNA was subsequently bisulfite treated (Epitect Bisulfite Kit, Qiagen, Inc., Valencia, CA). Following bisulfite treatment, DNA was then processed using direct bisulfite sequencing PCR following an established lab protocol to examine *Bdnf* methylation at exon *IV*^2,20,80^. Samples were sent to the University of Delaware Sequencing and Genotyping Center for sequencing using reverse primers. For each CG site (i.e. 1–11 of *Bdnf* exon *IV*), percent methylation was calculated using the ratio between peak values of G and A (G/[G + A]) on chromatograms using Chromas software.

The same DNA used for locus-specific analyses was used to assess global methylation. MethylFlash™ Methylated DNA Quantification Kits were used to quantify levels of genome-wide methylation (5-mC) according to the manufacturer’s instructions (Epigentek, Brooklyn, NY). Absorbance was measured using the Infinite ® F50 microplate reader (Tecan, Männedorf, Switzerland) with the amount of 5-mC DNA proportional to the intensity of the optical density. Samples were run in vertical duplicates at a strict concentration of 100 ng/well with total volume added per well not ranging outside of 2–5 ul per well.

### Gene expression

Gene expression was examined for a subset of genes using a previously established protocol^2,53^. The genes selected due to their role in processing rewarding stimuli, including the rewarding properties of pup interactions, included dopamine receptor 1 (*Drd1*) and µ-opioid receptor 1 (*Oprm1*)^37,47,50,71,117^. Genes selected due to their role in the stress response included corticotropin releasing factor receptor type 1 (*Crfr1*) and glucocorticoid receptor (*GR*)^5,118–122^. The plasticity-related genes selected were *Bdnf* (exons *I*, *IV*, and *IX*) and *fos*^123,124^. Estrogen receptor alpha (*ERα*) and oxytocin receptor (*Oxtr*) genes were assayed due to their role in maternal behavior onset^29,47,49,125^. The DNA methyltransferases (DNMTs) *DNMT1* and *DNMT3a* were probed to establish levels of epigenetic regulators. RNA taken from the brains of dams was subjected to a reverse-transcription reaction using a cDNA synthesis kit (Qiagen QuantiTect Reverse Transcription Kit). Subsequently, real-time PCR (Bio-Rad CFX96) was conducted using Taqman probes on cDNA (Applied Biosystems). PCR reactions were run in duplicates, and the results were averaged. *Tubulin* expression was used as a reference gene. Confirming stability of tubulin across our conditions, we did not find an effect of infant condition (*F*_(1,64)_ = 1.135, *p* = 0.2908), drug treatment (*F*_(1,64)_ = 0.04825, *p* = 0.8268), nor an interaction effect (*F*_(1,64)_ = 0.247, *p* = 0.6209). Gene expression was calculated for target genes relative to *tubulin* using the 2^−ΔΔ*C*T^ method^126^.

### Statistical analyses

Data were analyzed using GraphPad Prism. Behavioral data collected from the infant manipulations were analyzed using a one-way ANOVA. Behavioral (i.e. total number of adverse maternal behaviors in the 30 minute recording), DNA methylation, and gene expression data from dams previously exposed to infant manipulations were analyzed using two-way ANOVAs. T-tests were used for post-hoc analyses to further probe statistically significant effects, with Bonferroni corrections applied when necessary to reduce the chance of type I errors^127^. For all analyses, *p* < 0.05 was used as a threshold for statistical significance. Marginal significance was set at *p* < 0.06.
